# Damage Detection of Structures Identified with Deterministic-Stochastic Models Using Seismic Data

**DOI:** 10.1155/2014/879341

**Published:** 2014-08-03

**Authors:** Ming-Chih Huang, Yen-Po Wang, Ming-Lian Chang

**Affiliations:** ^1^Department of Aircraft Engineering, Air Force Institute of Technology, Gangshan 820, Taiwan; ^2^Department of Civil Engineering, National Chiao Tung University, Hsinchu 300, Taiwan

## Abstract

A deterministic-stochastic subspace identification method is adopted and experimentally verified in this study to identify the equivalent single-input-multiple-output system parameters of the discrete-time state equation. The method of damage locating vector (DLV) is then considered for damage detection. A series of shaking table tests using a five-storey steel frame has been conducted. Both single and multiple damage conditions at various locations have been considered. In the system identification analysis, either full or partial observation conditions have been taken into account. It has been shown that the damaged stories can be identified from global responses of the structure to earthquakes if sufficiently observed. In addition to detecting damage(s) with respect to the intact structure, identification of new or extended damages of the as-damaged counterpart has also been studied. This study gives further insights into the scheme in terms of effectiveness, robustness, and limitation for damage localization of frame systems.

## 1. Introduction

Structural health monitoring of civil engineering structures has received considerable attention over the last two decades with the progress in digital signal processing and system identification techniques [1–6]. Damage to a structure alters its dynamic characteristics in terms of the modal frequencies, damping ratios, and mode shapes, so as the physical parameters in terms of the stiffness or flexibility matrices. Despite that damage of a structure might be revealed from the changes of frequencies, it is difficult to locate the damages only with this information. The mode shapes perhaps provide a preferable basis for damage detection as they are spatially specific and reflective of local structural behavior. However, information of mode shapes alone is not sensitive enough for damage localization from the global dynamic vibration of the structure. The physical parameters indeed are more useful as far as damage localization is concerned. The study by Zhao and DeWolf [7] comparing various vibrational parameters for damage detection of spring-mass systems concluded that the model flexibilities were more sensitive to structural damages than either the natural frequencies or mode shapes. Stiffness is intuitively the most direct physical parameter related to damages in structures. However, the sensitivity analysis developed for damage detection based on stiffness matrix requires an accurate analytical model of the intact structure as a reference. Unfortunately, obtaining such an analytical model is in itself a difficult task. Moreover, synthesis of the stiffness matrix requires contributions of the higher modes that are practically difficult to identify. In contrast, the flexibility matrix can be sufficiently synthesized with a limited number of the lower modes as the modal contribution to the flexibility matrix decreases with the square of the corresponding modal frequency. Therefore, flexibility-based techniques have been considered of great potential in damage localization of structures from the global dynamic responses.

Another branch on the development of SHM techniques based on wavelet transformation has also gained a great deal of progress recently. A review by Li et al. [8] gives an insight of the up-to-date innovations on the SHM of infrastructures. In this paper, related theories and methodologies including sensing technology, sensor placement, signal processing, data fusion, system identification, and damage detection have been thoroughly discussed. Li et al. [9] propose a novel wavelet approach integrated with an auto-regressive moving average (ARMA) model for the damage detection of structures with progressive damage in time. The methodology has been validated both numerically and experimentally through shaking table tests of a reinforced concrete frame. The concept of wavelet transformation has also been adopted by Li et al. [10] to develop a method for determining the critical incidence of the seismic wave interpreted via energy principles. Yi et al. [11] proposes an energy-based multistage structural damage diagnosis method by adopting the schemes of wavelet packet transform (WPT) and artificial neural network (ANN). Using the benchmark structure of the American Society of Civil Engineers (ASCE) as the target, the authors demonstrate via numerical simulations that the proposed method is able to detect structural damage of various extents. Yet, localization of damages requires other measures.

Among the damage localization techniques based on variation of the physical parameters as the structure deteriorates, the flexibility-based approaches have been shown to be very promising and computationally efficient. Pandey and Biswas [12] demonstrated that the damage locations of a wide-flange steel beam could be successfully identified by interrogating the change in the flexibility. This technique has been further extended for damage detection of the plane trusses [13]. The flexibility-based damage localization technique referred to as the method of damage locating vectors (DLV) has been advanced further by Bernal [14] and Bernal and Gunes [15]. This methodology has also been adopted for damage localization of space trusses or plates by Gao et al. [16, 17] and Huynh et al. [18]. The concept of the DLV method is to identify the members with zero stress under some specific loading patterns, namely, the DLVs that span the null space of the change in flexibility matrix of the structure before and after the damage state. The loading vectors are obtained by performing the singular value decomposition (SVD) on the flexibility differential matrix. Those structural elements resulting with zero stresses (or internal forces) under the static loads of the DLVs are considered potentially damaged. The DLV technique is capable of identifying scenarios of multiple damages in the structure via a truncated modal basis without a predetermined reference model. This null space approach has recently been further extended by Bernal [19, 20] as the dynamic damage locating vector (DDLV) theorem that connects changes in the transfer matrices to the spatial location of stiffness related damage. Bernal [21] revised the DLV method further in the context of linear state-space models to comply with the stochastic realization of the systems by acknowledging that the DLVs in the null space of the flexibility differential can be extracted without a priori knowledge of the flexibility matrix. This scheme is adopted in this study.

System identification problems are commonly concerned with determining, from the relation of input/output sequences, the parameters of either polynomial models (e.g., ARMA, auto-regressive model with external input (ARX)) using the prediction error method [1] or state-space models by subspace system identification (SSI) [22], numerical algorithms for subspace state space system identification (N4SID) [23] for deterministic/stochastic systems, or SRIM for deterministic systems [24]. In contrast to the prediction error method that identifies the system parameters by minimizing the errors between the observations and those predicted by the model in a least squares sense, the stochastic realization theory initiated by Akaike [25] and Faure [26] is not based on the concept of optimization and leads to noniterative, convergence-guaranteed and efficient numerical algorithms. The covariance matrix is first constructed from block Hankle matrix [27] of shifted process sequences. The state-space model is in turn realized from the observability and/or controllability matrix via singular value decomposition of the covariance matrix. The theory of covariance-driven subspace method has been unified by van Overschee and de Moor [23] for deterministic, stochastic, and combined systems by defining the estimated state sequences as the projection of input-output data. The projected state sequences turn out to be the outputs of non-steady-state Kalman filter banks. To facilitate implementation of the DLV method, system identification technique of subspace technique or SRIM that identifies the system matrix and output (observation) matrix of a state-space model is considered a preferable alternative. The method of* system realization using information matrix* (*SRIM*) proposed by Juang [24] is based on a deterministic state-space system. The equivalent system matrix is identified from the covariance matrix of the input and output signals. This simple and elegant approach works well for systems in response to transient excitation if the noise level is negligible. The performance degrades, however, as the noise becomes pronounced. On the contrary, the* stochastic subspace identification* (*SSI*) method based on a stochastic model [23] without knowing the input is less sensitive to noise and works well if the excitation is Gaussian white noise. This scheme is not sufficient, however, for systems under transient excitations such as earthquakes. Therefore, a mixed deterministic-stochastic model is considered more robust to transient systems with nonnegligible noise contamination.

In this study, the feasibility of DLV method for damage detection of frame structures using seismic response data is explored in association with the subspace identification algorithm developed by van Overschee and de Moor [23] for mixed deterministic-stochastic systems. As an effort to verify the robustness of the deterministic-stochastic model against noise, system identification analysis by the N4SID algorithm is conducted with various noise levels and compared with those obtained by the SRIM. The effectiveness of the DLV method for damage detection using system parameters identified from contaminated signals is further investigated. Moreover, a series of shaking table tests using a five-storey steel frame has been conducted in National Center for Research on Earthquake Engineering (NCREE), Taiwan. Damage condition is simulated by reducing the cross-sectional area of some of the columns at the bottom. Both single and combinations of multiple damage conditions at various locations have been considered. In the system identification analysis, either full or partial observation conditions have been taken into account. It has been shown that the damaged stories can be identified from global responses of the structure to earthquakes if sufficiently observed. In addition to detecting damage(s) with respect to the intact structure, identification of new or extended damages of the as-damaged (ill-conditioned) counterpart has also been studied. The proposed scheme proves to be effective. This study gives further insights into the scheme in terms of effectiveness, robustness, and limitation for damage localization of frame systems.

## 2. Deterministic-Stochastic Subspace System Identification

A deterministic-stochastic linear time-invariant system is represented in a discrete-time state-space model as

(1) $$\begin{array}{c} z_{k+1}=Az_{k}+{Bu}_{k}+w_{k},\\ y_{k}=Cz_{k}+Du_{k}+v_{k} \end{array}$$

with

(2) $$\begin{array}{c} E\left[\left(\begin{array}{c} w_{k+\tau }\\ v_{k+\tau } \end{array}\right)\left(\begin{array}{cc} w_{k}^{T} & v_{k}^{T} \end{array}\right)\right]=\left[\begin{array}{cc} R_{ww}\left(\tau \right) & R_{wv}\left(\tau \right)\\ R_{wv}^{T}\left(\tau \right) & R_{vv}\left(\tau \right) \end{array}\right]\delta \left(\tau \right), \end{array}$$

where **z**_*k*_ ∈ *R* ^2*n*×1^ and **y**_*k*_ ∈ *R* ^*m*×1^ are, respectively, the state and output vectors and **u**_*k*_ ∈ *R* ^*r*×1^ is the input vector at time instant *k* with *n*, *m*, and *r* being, respectively, the dimension of the dynamic system, observational outputs, and external inputs. **A** ∈ *R* ^2*n*×2*n*^ is the system matrix, **B** ∈ *R* ^2*n*×*r*^ is the input influence matrix, **C** ∈ *R* ^*m*×2*n*^ is the observation matrix, and **D** ∈ *R* ^*m*×*r*^ is the direct transmission matrix. **w**_*k*_ ∈ *R* ^2*n*×1^ and **v**_*k*_ ∈ *R* ^*m*×1^ are the unmeasurable vector signals assumed to be zero-mean, stationary white noise vector sequences. *δ*[*τ*] is the Kronecker *δ*.

The state and output vectors are split into deterministic and stochastic parts as **z**_*k*+1_ = **z**_*k*+1_^*d*^ + **z**_*k*+1_^*s*^ and **y**_*k*_ = **y**_*k*_^*d*^ + **y**_*k*_^*s*^ satisfying, respectively, the deterministic subsystem

(3) zk+1d=Azkd+Buk,ykd=Czkd+Duk

and the stochastic subsystem

(4) zk+1s=Azks+wk,yks=Czks+vk.

By recursive substitution into the state-space equations of consecutive shifted processes, it leads to [28]

(5) Y0 ∣ i−1=ΓiZ0d+HiU0 ∣ i−1+Y0 ∣ i−1s,Yi ∣ 2i−1=ΓiZid+HiUi ∣ 2i−1+Yi ∣ 2i−1s,Zid=AiZ0d+ΛiU0 ∣ i−1,

where

(6) $$\begin{array}{c} Y_{0\;|\;i-1}=\left[\begin{array}{ccccc} y_{0} & y_{1} & y_{2} & \cdots & y_{j-1}\\ y_{1} & y_{2} & y_{3} & \cdots & y_{j}\\ y_{2} & y_{3} & y_{4} & \cdots & y_{j+1}\\ \vdots & \vdots & \vdots & \vdots & \vdots \\ y_{i-1} & y_{i} & y_{i+1} & \cdots & y_{i+j-2} \end{array}\right]\in R^{mi\times j} \end{array}$$

is the output block Hankle matrix [27] of the past where *i* is the number of steps to be considered in the Markov process and *j* represents the length of the data to be included in the analysis;

(7) $$\begin{array}{c} U_{0\;|\;i-1}=\left[\begin{array}{ccccc} u_{0} & u_{1} & u_{2} & \cdots & u_{j-1}\\ u_{1} & u_{2} & u_{3} & \cdots & u_{j}\\ u_{2} & u_{3} & u_{4} & \cdots & u_{j+1}\\ \vdots & \vdots & \vdots & \vdots & \vdots \\ u_{i-1} & u_{i} & u_{i+1} & \cdots & u_{i+j-2} \end{array}\right]\in R^{ri\times j} \end{array}$$

is the input block Hankle matrix of the past;

(8) $$\begin{array}{c} Y_{0\;|\;i-1}^{s}=\left[\begin{array}{ccccc} y_{0}^{s} & y_{1}^{s} & y_{2}^{s} & \cdots & y_{j-1}^{s}\\ y_{1}^{s} & y_{2}^{s} & y_{3}^{s} & \cdots & y_{j}^{s}\\ y_{2}^{s} & y_{3}^{s} & y_{4}^{s} & \cdots & y_{j+1}^{s}\\ \vdots & \vdots & \vdots & \vdots & \vdots \\ y_{i-1}^{s} & y_{i}^{s} & y_{i+1}^{s} & \cdots & y_{i+j-2}^{s} \end{array}\right]\in R^{mi\times j} \end{array}$$

is the stochastic output block Hankle matrix of the past;

(9) $$\begin{array}{c} Z_{i}^{d}=\left[\begin{array}{ccccc} z_{i}^{d} & z_{i+1}^{d} & z_{i+2}^{d} & \cdots & z_{i+j-1}^{d} \end{array}\right]\in R^{2n\times j} \end{array}$$

is the deterministic state matrix;

(10) $$\begin{array}{c} \Gamma_{i}=\left[\begin{array}{c} C\\ CA\\ CA^{2}\\ \vdots \\ CA^{i-1} \end{array}\right]\in R^{mi\times 2n} \end{array}$$

is the observability matrix;

(11) $$\begin{array}{c} \Lambda_{i}=\left[\begin{array}{ccccc} A^{i-1}B & A^{i-2}B & \cdots & AB & B \end{array}\right]\in R^{2n\times ri} \end{array}$$

is the controllability matrix;

(12) $$\begin{array}{c} H_{i}=\left[\begin{array}{ccccc} D & 0 & 0 & \cdots & 0\\ CB & D & 0 & \cdots & 0\\ CAB & CB & D & \cdots & 0\\ \vdots & \vdots & \vdots & \vdots & \vdots \\ CA^{i-2}B & CA^{i-3}B & CA^{i-4}B & \cdots & D \end{array}\right]\in R^{mi\times ri} \end{array}$$

is the triangular Toeplits matrix [27], and

(13) $$\begin{array}{c} A_{i}=\left[\begin{array}{c} A\\ A^{2}\\ A^{3}\\ \vdots \\ A^{i} \end{array}\right]\in R^{2ni\times 2n}. \end{array}$$

The stochastic covariance equations of the subspace can be defined as

(14) Ps=E[zks(zks)T]=APsAT+Rww(0),G=E[zk+1s(yks)T]=APsCT+Rwv(1),λ0=E[yks(yks)T]=CPsCT+Rvv(0)

in which **P** ^*s*^ and ***λ***_0_ represent, respectively, the auto-covariance matrix of the state (**z**_*k*_^*s*^) and output (**y**_*k*_^*s*^) vectors and **G** is the covariance matrix of the state (**z**_*k*+1_^*s*^) and output (**y**_*k*_^*s*^) vectors.

The block Toeplitz covariance matrix Λ_0∣*i*−1_^*s*^ of the stochastic output is defined as

(15) Λ0 ∣ i−1s=1NY0 ∣ i−1Y0 ∣ i−1T=[λ0λ−1λ−2⋯λ1−iλ1λ0λ−1⋯λ2−iλ2λ1λ0⋯λ3−i⋮⋮⋮⋮⋮λi−1λi−2λi−3⋯λ0]∈Rmi×mi,

where

(16) $$\begin{array}{c} \lambda_{i}=E\left[\begin{array}{cc} y_{k+i}^{s} & {\left(y_{k}^{s}\right)}^{T} \end{array}\right]=\{\begin{array}{cc} CA^{i-1}G & i>0\\ \lambda_{0} & i=0\\ G^{T}{\left(A^{T}\right)}^{-i-1}C^{T} & i<0 \end{array} \end{array}$$

and the block Toeplitz cross covariance matrix Λ_*i*∣2*i*−1_^*s*^ is defined similarly as

(17) Λi ∣ 2i−1s=1NYi ∣ 2i−1Y0 ∣ i−1T=[λiλi−1λi−2⋯λ1λi+1λiλi−1⋯λ2λi+2λi+1λi⋯λ3⋮⋮⋮⋮⋮λ2i−1λ2i−2λ2i−3⋯λi]=ΓiΛis∈Rmi×mi,

where

(18) $$\begin{array}{c} \Lambda_{i}^{s}=\left[\begin{array}{ccccc} A^{i-1}G & A^{i-2}G & A^{i-3}G & \cdots & G \end{array}\right]\in R^{2n\times mi} \end{array}$$

and *N* represent the data length. Equation (18) is the stochastic controllability matrix. Projecting the future output state matrix **Y**_*i*∣2*i*−1_ onto the input matrix **U**_0∣2*i*−1_ and the past output state matrix **Y**_0∣*i*−1_, the output-input block Hankel matrices are constructed as

(19) Y−i=Yi ∣ 2i−1(U0 ∣ 2i−1Y0 ∣ i−1)=Yi ∣ 2i−1(U0 ∣ 2i−1Y0 ∣ i−1)T((U0 ∣ 2i−1Y0 ∣ i−1)(U0 ∣ 2i−1Y0 ∣ i−1)T)∗ ×(U0 ∣ 2i−1Y0 ∣ i−1),

(20) Y−i+1=Yi+1 ∣ 2i−1(U0 ∣ 2i−1Y0 ∣ i)=Yi+1 ∣ 2i−1(U0 ∣ 2i−1Y0 ∣ i)T((U0 ∣ 2i−1Y0 ∣ i)(U0 ∣ 2i−1Y0 ∣ i)T)∗ ×(U0 ∣ 2i−1Y0 ∣ i),

where (·)* denotes the pseudoinverse of the matrix (·). To further simplify (19) and (20), the deterministic subspace and stochastic subspace are utilized to define what follows:

(21) lim⁡j→∞⁡1j(U0 ∣ i−1Ui ∣ 2i−1Z0d)(U0 ∣ i−1T ∣ Ui ∣ 2i−1T ∣ (Z0d)T) =[R11R12S1TR12TR22S2TS1S2Pd] =[RSTSPd]∈R(2ri+2n)×(2ri+2n),lim⁡j→∞⁡1j(Y0 ∣ i−1sYi ∣ 2i−1s)((Y0 ∣ i−1s)T ∣ (Yi ∣ 2i−1s)T) =(Λ0 ∣ i−1s(Λi ∣ 2i−1s)TΛi ∣ 2i−1sΛ0 ∣ i−1s).

Assuming that the deterministic input **u**_*k*_ and the deterministic state **z**_*k*_^*d*^ are independent of the stochastic output **y**_*k*_^*s*^, then (19) and (20) can be simplified as

(22) $$\begin{array}{c} {\bar{Y}}_{i}=\Gamma_{i}{\hat{Z}}_{i}+H_{i}U_{i\;|\;2i-1},\\ {\bar{Y}}_{i+1}=\Gamma_{i-1}{\hat{Z}}_{i+1}+H_{i-1}U_{i+1\;|\;2i-1}, \end{array}$$

where

(23) Z^i=(Ai−QiΓi ∣ Λi−QiHi ∣ Qi)(SR−1U0 ∣ 2i−1U0 ∣ i−1Y0 ∣ i−1),Z^i+1=(Ai+1−Qi+1Γi+1 ∣ Λi+1−Qi+1Hi+1 ∣ Qi+1) ×(SR−1U0 ∣ 2i−1U0 ∣ iY0 ∣ i),

where

(24) $$\begin{array}{c} Q_{i}=x_{i}\psi_{i}^{-1} \end{array}$$

in which

(25) xi=Ai(Pd−SR−1ST)ΓiT+Λis,ψi=Γi(Pd−SR−1ST)ΓiT+Λ0 ∣ i−1s.

It follows immediately from (22) that

(26) $$\begin{array}{c} {\hat{Z}}_{i}=\Gamma_{i}^{*}\left({\bar{Y}}_{i}-H_{i}U_{i\;|\;2i-1}\right),\\ {\hat{Z}}_{i+1}=\Gamma_{i-1}^{*}\left({\bar{Y}}_{i+1}-H_{i-1}U_{i+1\;|\;2i-1}\right), \end{array}$$

where Γ_i_* or Γ_*i*−1_* denotes the pseudoinverse of its counterpart. The sequences ${\hat{Z}}_{i}$ and ${\hat{Z}}_{i+1}$ proved to be the states of a bank of *j* non-steady-state Kalman filters [23]. The non-steady-state Kalman filter state ${\hat{Z}}_{k}$ can be defined in a recursive form as [23]

(27) z^k=Az^k−1+Buk−1+Kk−1(yk−1−Cz^k−1−Duk−1),

(28) Kk−1=(APk−1CT+G)(λ0+CPk−1CT)−1,

(29) Pk=APk−1AT−Kk−1(APk−1CT+G)T.

If we collect consecutive *j* columns of the Kalman states in parallel, (27) can then be extended as

(30) Z^i+1=AZ^i+BUi ∣ i+Ki(Yi ∣ i−CZ^i−DUi ∣ i)=AZ^i+BUi ∣ i+Wi ∣ i,

where $W_{i|i}=\left(\begin{array}{ccccc} w_{i} & w_{i+1} & w_{i+2} & \cdots & w_{i+j-1} \end{array}\right)$. It is trivial that

(31) Yi ∣ i=CZ^i+DUi ∣ i+(Yi ∣ i−CZ^i−DUi ∣ i)=CZ^i+DUi ∣ i+Vi ∣ i,

where $V_{i|i}=\left(\begin{array}{ccccc} v_{i} & v_{i+1} & v_{i+2} & \cdots & v_{i+j-1} \end{array}\right)$. Combining (30) and (31), one gets

(32) $$\begin{array}{c} \left(\begin{array}{c} {\hat{Z}}_{i+1}\\ Y_{i\;|\;i} \end{array}\right)=\left(\begin{array}{c} A\\ C \end{array}\right){\hat{Z}}_{i}+\left(\begin{array}{c} B\\ D \end{array}\right)U_{i\;|\;i}+\left(\begin{array}{c} W_{i\;|\;i}\\ V_{i\;|\;i} \end{array}\right). \end{array}$$

Substituting (26) for ${\hat{Z}}_{i}$ and ${\hat{Z}}_{i+1}$ into (32), it leads to

(33) $$\begin{array}{c} \left(\begin{array}{c} \Gamma_{i-1}^{*}{\bar{Y}}_{i+1}\\ Y_{i\;|\;i} \end{array}\right)=\left(\begin{array}{cc} A & K_{12}\\ C & K_{22} \end{array}\right)\left(\begin{array}{c} \Gamma_{i}^{*}{\bar{Y}}_{i}\\ U_{i\;|\;2i-1} \end{array}\right)+\left(\begin{array}{c} W_{i\;|\;i}\\ V_{i\;|\;i} \end{array}\right) \end{array}$$

in which

(34) K12=(B−AΓi∗(DΓi−1B) ∣ Γi−1∗Hi−1−AΓi∗(0Hi−1))∈Rn×mi,K22=(D−CΓi∗(DΓi−1B) ∣ −CΓi∗(0Hi−1))∈Rl×mi.

By forcing the noise terms in (33) to be zero, the coefficient matrices may be resolved as

(35) $$\begin{array}{c} \left(\begin{array}{cc} A & K_{12}\\ C & K_{22} \end{array}\right)=\left(\begin{array}{c} \Gamma_{i-1}^{*}{\bar{Y}}_{i+1}\\ Y_{i\;|\;i} \end{array}\right){\left(\begin{array}{c} \Gamma_{i}^{*}{\bar{Y}}_{i}\\ U_{i\;|\;2i-1} \end{array}\right)}^{*}, \end{array}$$

where ${\left(\begin{array}{c} \Gamma_{i}^{*}{\bar{Y}}_{i}\\ U_{i|2i-1} \end{array}\right)}^{*}$ is the pseudoinverse of $\left(\begin{array}{c} \Gamma_{i}^{*}{\bar{Y}}_{i}\\ U_{i|2i-1} \end{array}\right)$. A numerically stable and efficient algorithm referred to as the N4SID devised by van Overschee and de Moor [22] is adopted in this study to solve for the system matrix.

## 3. N4SID Algorithm

To facilitate implementation of the numerical algorithm, van Overschee and de Moor [22] proposed a numerically stable procedure N4SID utilizing the RQ decomposition of the block Hankle matrix

(36) $$\begin{array}{c} H=\frac{\left(\begin{array}{c} U_{0\;|\;2i-1}\\ Y_{0\;|\;2i-1} \end{array}\right)}{\sqrt{j}}=\hat{R}{\hat{Q}}^{T}, \end{array}$$

where $\hat{Q}{\hat{Q}}^{T}=I$ and $\hat{R}$ is a lower triangular matrix. It can be expressed in the following partitioned form as

(37) (U0 ∣ i−1Ui ∣ iUi+1 ∣ 2i‐1Y0 ∣ i−1Yi ∣ iYi+1 ∣ 2i‐1)=(R^1100000R^21R^220000R^31R^32R^33000R^41R^42R^43R^4400R^51R^52R^53R^54R^550R^61R^62R^63R^64R^65R^66) ×(Q^1TQ^2TQ^3TQ^4TQ^5TQ^6T)

which can be written in a more condensed manner as

(38) $$\begin{array}{c} \left(\begin{array}{c} U_{0\;|\;2i-1}\\ Y_{0\;|\;i-1}\\ Y_{i\;|\;2i-1} \end{array}\right)=\left(\begin{array}{cc} R_{11}^{i} & 0\\ R_{21}^{i} & R_{22}^{i} \end{array}\right)\left(\begin{array}{c} {\left(Q_{1}^{i}\right)}^{T}\\ {\left(Q_{2}^{i}\right)}^{T} \end{array}\right) \end{array}$$

or

(39) $$\begin{array}{c} \left(\begin{array}{c} U_{0\;|\;2i-1}\\ Y_{0\;|\;i}\\ Y_{i+1\;|\;2i-1} \end{array}\right)=\left(\begin{array}{cc} R_{11}^{i+1} & 0\\ R_{21}^{i+1} & R_{22}^{i+1} \end{array}\right)\left(\begin{array}{c} {\left(Q_{1}^{i+1}\right)}^{T}\\ {\left(Q_{2}^{i+1}\right)}^{T} \end{array}\right) \end{array}$$

with

(40) R11i=(R^11000R^21R^2200R^31R^32R^330R^41R^42R^43R^44);R21i=(R^51R^52R^53R^54R^61R^62R^63R^64);R22i=(R^550R^65R^66);(Q1i)T=(Q^1TQ^2TQ^3TQ^4T);  (Q2i)T=(Q^5TQ^6T),R11i+1=(R^110000R^21R^22000R^31R^32R^3300R^41R^42R^43R^440R^51R^52R^53R^54R^55);R21i+1=(R^61R^62R^63R^64R^65);R22i+1=(R^66);  (Q1i+1)T=(Q^1TQ^2TQ^3TQ^4TQ^5T),(Q2i+1)T=(Q^6T).

Now if we express $Y_{i|2i-1}=\left(\begin{array}{cc} R_{21}^{i} & R_{22}^{i} \end{array}\right){\hat{Q}}^{T}$ and $\left(\begin{array}{c} U_{0|2i‐1}\\ Y_{0|i‐1} \end{array}\right)=\left(\begin{array}{cc} R_{11}^{i} & 0 \end{array}\right){\hat{Q}}^{T}=$ **R**_11_^*i*^(**Q**_1_^*i*^)^*T*^, then (19) can be written as

(41) $$\begin{array}{c} {\overset{-}{Y}}_{i}=R_{21}^{i}{\left(R_{11}^{i}\right)}^{-1}\left(\begin{array}{c} U_{0\;|\;2i‐1}\\ Y_{0\;|\;i‐1} \end{array}\right)=\left(L_{1}^{i}\;|\;L_{2}^{i}\;|\;L_{3}^{i}\right)\left(\begin{array}{c} U_{0\;|\;i‐1}\\ U_{i\;|\;2i‐1}\\ Y_{0\;|\;i-1} \end{array}\right). \end{array}$$

By the same token, (20) may also be written as

(42) Y−i+1=R21i+1(R11i+1)−1(U0 ∣ 2i‐1Y0 ∣ i‐1)=(L1i+1 ∣ L2i+1 ∣ L3i+1)(U0 ∣ i‐1Ui+1 ∣ 2i‐1Y0 ∣ i).

It has been shown by van Overschee and de Moor [22] that the observability matrix Γ_*i*_ can be obtained from SVD of

(43) (L1i0L3i)(U0 ∣ i‐1Ui ∣ 2i‐1Y0 ∣ i−1)=(L1i0L3i)R11i(Q1i)T=(U1U2)(Σ100Σ2)((Q1i)TVT).

The rank is determined from the dominant singular values of the decomposition, and Γ_*i*_ can be determined as

(44) $$\begin{array}{c} \Gamma_{i}=U_{1}\Sigma_{1}^{1/2} \end{array}$$

and Γ_*i*−1_ can be extracted directly from Γ_*i*_ without the last *l* rows. With Γ_*i*_ and Γ_*i*−1_ determined, one can calculate $\Gamma_{i}^{*}{\bar{Y}}_{i}$ and $\Gamma_{i-1}^{*}{\bar{Y}}_{i+1}$ without difficulty and in turn solve (35).

## 4. Review of the DLV Method

Bernal and Gunes [15] proposed that the structure subjected to the damage locating vectors, **L**, would undergo the same deformation before and after the damaged state. This statement immediately leads to

(45) $$\begin{array}{c} D_{F}L=0, \end{array}$$

where **D**_*F*_ is the flexibility differential of the structure before and after being damaged. When rank (**D**_*F*_) < *n* (*n* is the degree of freedom of the structure), the basis corresponding to the null space of **D**_*F*_ is the damage locating vectors, **L**, which can be derived from singular value decomposition of the flexibility differential of the structure before and after the damage state. Members with nearly zero stress under the loadings of DLVs are considered potentially damaged.

The flexibility matrix of the structure can be expressed with the system matrices of the continuous-time state-space representation as

(46) $$\begin{array}{c} F=-C_{0}A_{c}^{-1}H^{-1}C_{0}^{T}\tilde{D}=Q\tilde{D}, \end{array}$$

where **A**_*c*_ = ln⁡(**A**)/Δ*t* ∈ *R* ^2*n*×2*n*^ is the continuous-time system matrix; $C_{0}=\left[\begin{array}{cc} I & 0 \end{array}\right]\in R^{n\times 2n}$; $H=\left[\begin{array}{c} C_{0}\\ C_{0}A_{c} \end{array}\right]\in R^{2n\times 2n}$; $\tilde{D}=C_{0}A_{c}B_{c}=-M^{-1}$ (**M** being the mass matrix of the system).

With (46), the flexibility differential **D**_*F*_ can be expressed as

(47) $$\begin{array}{c} D_{F}=\Delta Q{\tilde{D}}^{i}+Q^{d}\Delta \tilde{D}=\Delta Q{\tilde{D}}^{i}, \end{array}$$

where Δ**Q** = **Q** ^*d*^ − **Q** ^*i*^ and $\Delta \tilde{D}={\tilde{D}}^{d}-{\tilde{D}}^{i}=0$ since the mass matrix is unchanged. By taking the singular value decomposition of Δ**Q**, the eigenvectors **v**_0_^Δ**Q**^ corresponding to the singular eigenvalues are the damage locating vector **L** ∈ *R* ^*n*×*q*^.

The weighted stress index WSI_*j*_ is defined as

(48) $$\begin{array}{c} {\text{WSI}}_{j}=\overset{q}{\underset{i=1}{\sum }}{\text{nsi}}_{j,i}, \end{array}$$

where nsi_*j*,1_ is the normalized stress index of the *j*th member or d.o.f. subjected to the *i*th DLV. Member *j* (or storey *j*) is considered seriously damaged when the normalized weighted stress index nWSI_*j*_ ≤ 0.1 in which nWSI_*j*_/WSI_*j*,max⁡_, whereas it is considered moderately damaged as 0.1 < nWSI_*j*_ ≤ 0.2.

## 5. Numerical Illustration of the Proposed Schemes

**Figure 1:**
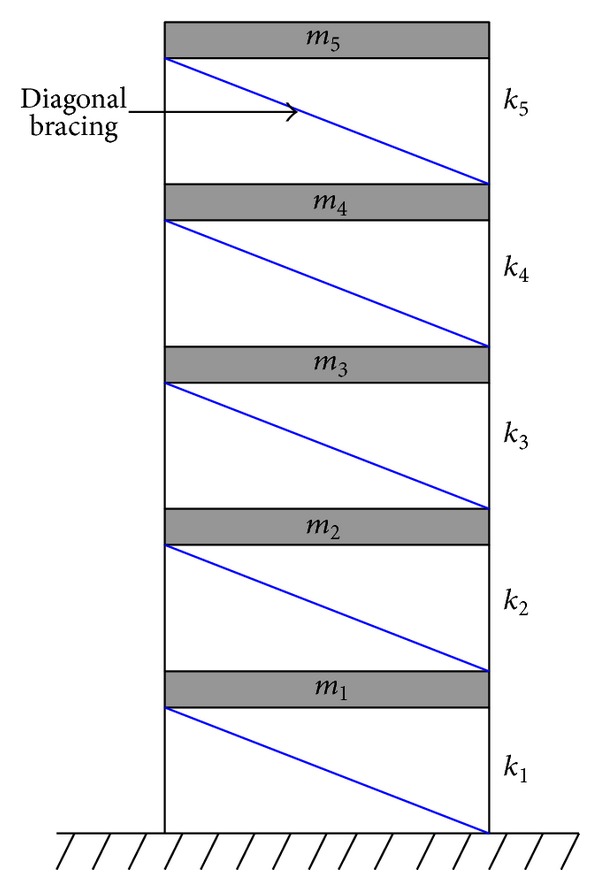
Illustrative diagram of a five-storey shear frame.

As an effort to verify the robustness of the deterministic-stochastic model against noise, system identification analysis by the N4SID algorithm is conducted with various noise levels and compared with those obtained by the SRIM. A five-story diagonally braced shear frame (Figure 1) with parameters shown in Table 1 is considered in the numerical example. The 1940 El Centro earthquake with peak ground acceleration (PGA) scaled to 0.1 g is considered as the input. To simulate noisy conditions, white noises of various noise-to-signal ratios (NSR) are added to the dynamic response signals. The noise-to-signal ratio at the *m*th storey, NSR_*m*_, is defined as

(49) $$\begin{array}{c} {\text{NSR}}_{m}=\frac{{\text{RMS}}_{N,m}}{{\text{RMS}}_{O,m}}\times 100\%, \end{array}$$

where RMS_*N*,*m*_ and RMS_*O*,*m*_ represent, respectively, the root mean squares (RMS) of the added white noise and the original response signals at the *m*th storey. In the simulation, the noise levels at all floors are assumed to be of the same amount in each case with NSR = 0%, 5% and 10%, respectively.

To assess the accuracy of the identification results, an error index (*E*_*i*_) of the *i*th mode is defined as

(50) $$\begin{array}{c} E_{i}=\sqrt{\frac{1}{n}\overset{n}{\underset{k=1}{\sum }}\varepsilon_{i,k}}, \end{array}$$

where *n* is the total number of modes, $\varepsilon_{i,k}={\left({\hat{\phi }}_{i,k}-\phi_{i,k}\right)}^{2}/{\hat{\phi }}_{i,k}^{2}$ in which ${\hat{\phi }}_{i,k}$ and *ϕ*_*i*,*k*_ are, respectively, the *k*th element of the *i*th mode shape derived from the eigen analysis and system identification.

**Figure 2:**
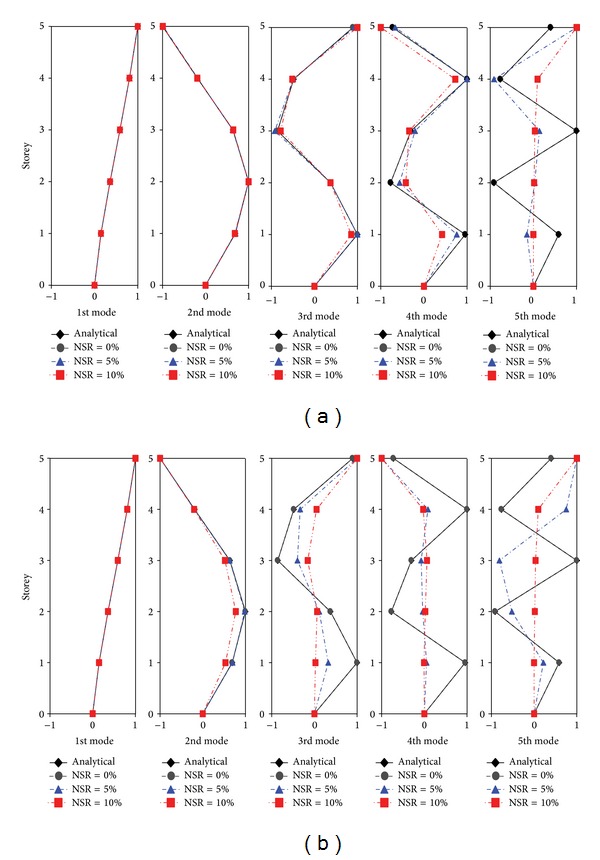
(a) Mode shapes identified with N4SID under various noise levels. (b) Mode shapes identified with SRIM under various noise levels.

**Figure 3:**
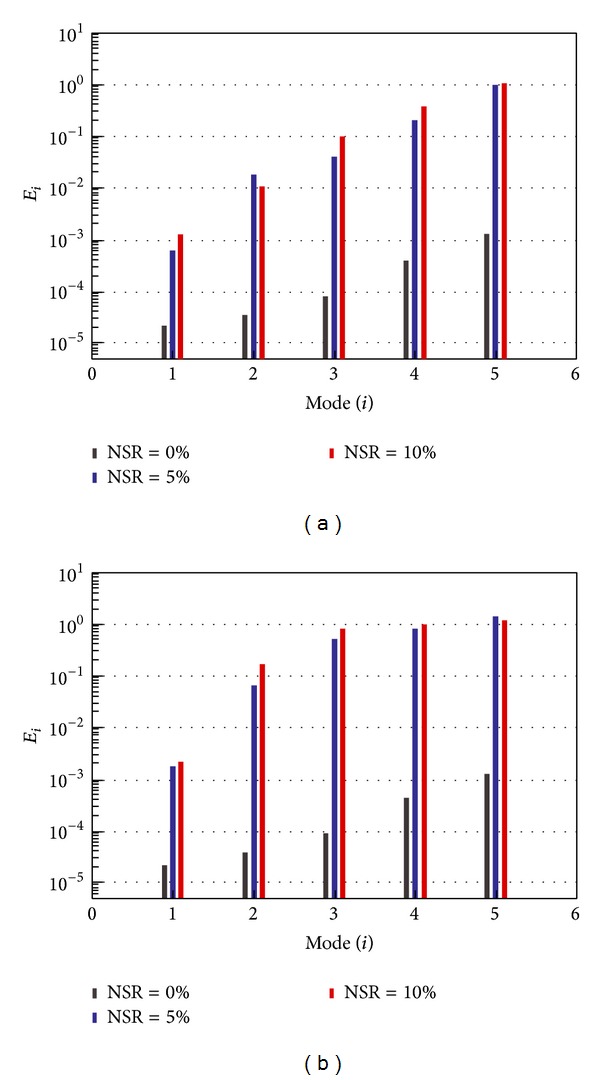
(a) Comparison of the error index with N4SID under various noise levels. (b) Comparison of the error index with SRIM under various noise levels.

Figure 2(a) shows the comparison of mode shapes identified by the N4SID algorithm at various NSR levels with the analytical solutions. When the signals are noise-free (NSR = 0%), all the identified mode shapes coincide with their corresponding analytical counterparts. Deviation of the mode shapes increases with the noise level and becomes more pronounced for higher modes as observed from Figure 2(a). This is also revealed quantitatively from the error index (*E*_*i*_) bar charts as illustrated in Figure 3(a). When the noise level achieves NSR = 10%, only the first three modes can be identified with acceptable accuracy. The results by the SRIM exhibit similar trends, as shown in Figures 2(b) and 3(b), but deviate in a larger extent from the analytical solutions. When the noise level achieves NSR = 10%, moreover, only the first mode is identified with fidelity. The N4SID algorithm proves more robust to noises than the SRIM, as expected.

In order to examine if the damage detection scheme works sufficiently when the dynamic response signals are contaminated with noises, numerical simulations are next conducted using the same analytical model. Damage conditions of the structure are simulated by removing some of the diagonal bracings. Both single and multiple damages have been considered, which includes Cases Dl, D2, D3, D4, D5, D13, D15, and D135 where the number(s) denote the damaged stories. Only the N4SID algorithm will be adopted and a noise level of NSR = 10% is considered. Analysis for the noise-free condition has also been carried out as a reference. The results are summarized and compared in Table 2. The shaded area in the table with the normalized weighted stress index nWSI_*j*_ < 0.2 indicates those identified as potentially damaged. It is observed that in both cases with NSR = 0% and 10%, the damaged stories can be successfully identified without exception, despite that results of the noise-free condition are shown to be more sensitive in the sense of getting smaller nWSI_*j*_  values at the damaged locations. The proposed scheme proves effective even if there exists a certain level of noise and only the first three modes are accurately identified.

## 6. Experimental Verifications

**Figure 4:**
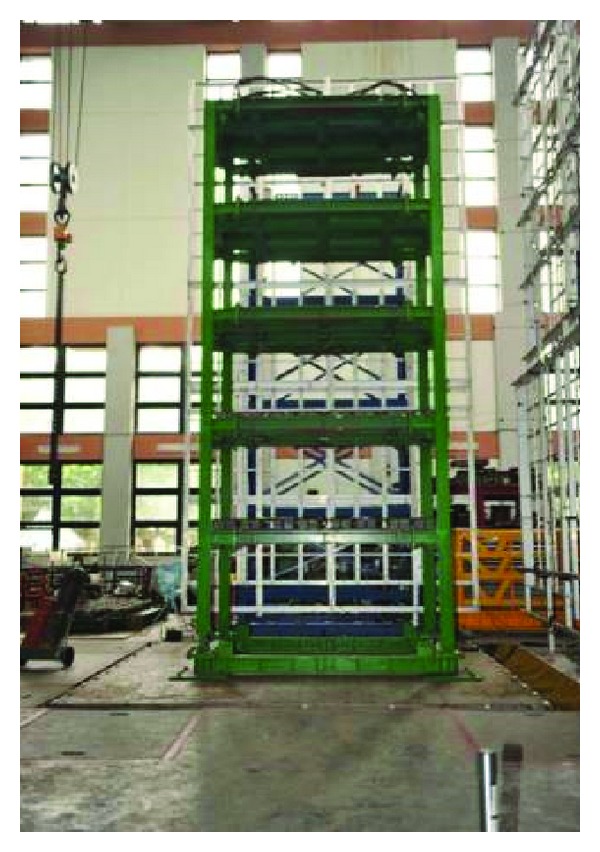
The benchmark model for experimental verification.

**Figure 5:**
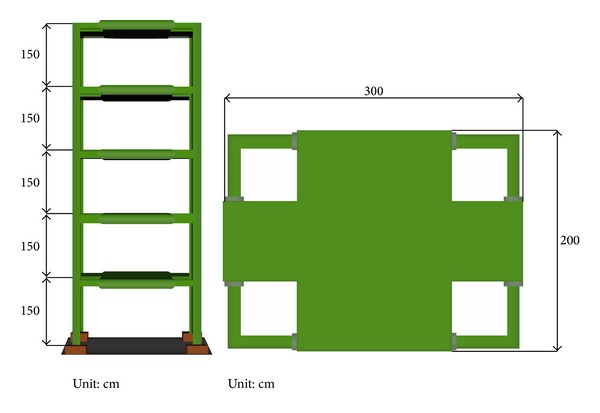
Elevation and layout of the benchmark model.

**Figure 6:**
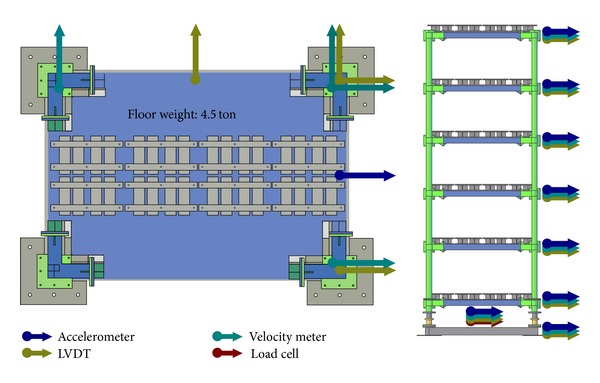
The sensor layout of the benchmark structure.

As a further step in verifying the feasibility of the proposed scheme experimentally, a series of shaking table tests has been carried out in the National Center for Research on Earthquake Engineering (NCREE), Taiwan, using a benchmark model (Figure 4). Weighing 22.5 tons, the benchmark model consisting of steel I-beams (*H*150 × 150 × 7 × 10) is a five-storey frame of 7.5 m in height and 3 m × 2 m in plane, as illustrated in Figure 5. Accelerometers have been implemented at each floor and the base to monitor the dynamic responses of the model to ground motion which together serve as the basis for system identification. In order to get more insight of the structural behavior, additional velocity meters, LVDT, and load cells have also been implemented in the tests, as illustrated in Figure 6. Only the acceleration information is utilized, however, in this study.

**Figure 7:**
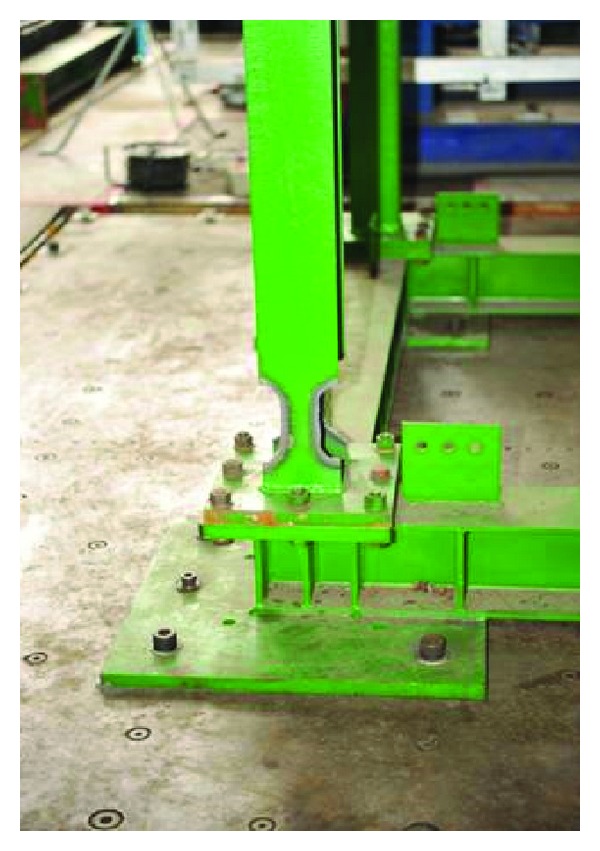
Flange partially cut out near the bottom of column.

Damage of the structure is simulated by cutting out a small portion of the flange near the bottom of the column(s), as shown in Figure 7. In order to sufficiently examine the damage at various extents, the damages were progressively enforced on one side of the frame from the first storey up to the third storey from one test to another. It is meant to represent a moderate damage condition as a single column is damaged in the same storey while representing a serious damage condition as two columns are damaged. In total, six damage conditions have been considered in the test program. The 1940 El Centro earthquake has been adopted as the input with the PGA scaled to 0.1 g.

The test results are analyzed under considerations of full observation (utilizing acceleration responses of all stories), partial observation (ignoring accelerations at some stories), and the ill-conditioned condition where the reference structure has been wounded in previous tests.

### 6.1. Full Observation

The six damaged conditions simulated in the tests are designated as:

M1: single column damaged at the first storey, representing a moderate damage condition of the first storey;
S1: two columns damaged at the first storey, representing a serious damage condition of the first storey;
S1M2: two columns damaged at the first storey and single column damaged at the second storey;
S12: two columns damaged at both the first and second stories;
S12M3: two columns damaged at both the first and second stories and single column damaged at the third storey;
S123: two columns damaged from storey 1 to 3.

The assessment results of various damage conditions based on full observation data of the structure are summarized in Table 3 where the shaded area corresponds to those being screened out as potentially damaged stories. Take case M1 for example, only the normalized weighted stress index of 1F is below 0.1, indicating damage of the first storey. This agrees with the actual damage condition. And in case S1M2, the index is 0.04 for 1F and 0.12 for 2F, agreeing with the actual condition that the first storey is seriously damaged and the second storey is moderately damaged. It is evident that, under a full observation condition, the damaged location(s) are successfully identified, regardless of single or multiple damage conditions.

### 6.2. Partial Observation

The 12 cases analyzed in a partial observation condition are designated below as follows:

 M1/135: single column damaged at the first storey, representing a moderate damage condition of the first storey with only the first, third, and fifth floors observed;
 S1/135: two columns damaged at the first storey, representing a serious damage condition of the first storey with only the first, third, and fifth floors observed;
 M1/124: single column damaged at the first storey with only the first, second, and fourth floors observed;
 S1/124: two columns damaged at the first storey with only the first, second, and fourth floors observed;
 S1M2/124: two columns damaged at the first storey and single column damaged at the second storey with only the first, second, and fourth floors observed;
 S1M2/125: two columns damaged at the first storey and single column damaged at the second storey with only the first, second, and fifth floors observed;
 S1M2/1235: two columns damaged at the first storey and single column damaged at the second storey without observing the fourth floor;
 S12/124: two columns damaged at both the first and second stories with only the first, second, and fourth floors observed;
 S12/125: two columns damaged at both the first and second stories with only the first, second, and fifth floors observed;
 S12/1235: two columns damaged at both the first and second stories without observing the fourth floor;
 S12M3/1235: two columns damaged at both the first and second stories and single column damaged at the third storey without observing the fourth floor;
 S123/1235: two columns damaged at the first, second and third stories without observing the fourth floor.

The assessment results of various damage conditions based on partial observation data of the structure are summarized in Table 4 where the shaded area corresponds to those being screened out as potentially damaged stories. The performance index (PMC) is similarly defined as in Table 3. The results show that the scheme with partial observation remains effective for single damage conditions, as in the cases of M1/135, S1/135, M1/124 and S1/124 where the index corresponding to the first storey is below 0.2. The scheme fails, however, to locate the damaged stories in multiple damage conditions except for case S12/1235 where the first two stories are seriously damaged and 4 out of 5 stories are observed.

### 6.3. Ill-Conditioned Structures

At times the structural health monitoring system might be introduced after the target building has been previously damaged. It is of interest to verify if the scheme is able to identify new or extended damage(s) in an earthquake event of an ill-conditioned structure that has been previously damaged. The system identification analysis will be based on full observation data as it provides more reliable structural information for damage assessment. The 10 cases considered for damage detection analysis of ill-conditioned structures are designated below as follows:

 S1/M1: structure seriously damaged at the first storey of the current state versus the one with its first storey moderately damaged earlier;
 S1M2/S1: structure damaged seriously at the first storey and moderately at the second storey of the current state versus the one with its first storey seriously damaged earlier;
 S12/S1M2: structure damaged seriously at both the first and second stories of the current state versus the one seriously damaged at its first storey and moderately at the second in advance;
 S12M3/S12: structure damaged seriously at both the first and second stories and moderately at the third storey of the current state versus the one with its both first and second stories seriously damaged earlier;
 S123/S12M3: structure seriously damaged at the first, second, and third stories of the current state versus the one seriously damaged at its both first and second stories and moderately at the third in advance;
 S12/M1: structure seriously damaged at both the first and second stories of the current state versus the one with its first storey moderately damaged earlier;
 S12M3/M1: structure seriously damaged at both the first and second stories and moderately at the third of the current state versus the one with its first storey moderately damaged earlier;
 S123/M1: structure seriously damaged at the first, second, and third stories of the current state versus the one with its first storey moderately damaged earlier;
 S12/S1: structure seriously damaged at both the first and second stories of the current state versus the one with its first storey seriously damaged earlier;
 S123/S1: structure seriously damaged at the first, second, and third stories of the current state versus the one with its first storey seriously damaged earlier.

The assessment results of various damage conditions in respect to an ill-conditioned structure damaged earlier are summarized in Table 5. The performance index (PMC) is similarly defined as in Table 3. In all the cases considered, the scheme proves sufficient in identifying new or extended damage(s) without exception under a full observation condition in the system identification process, as indicated from the indices.

## 7. Summary and Conclusion

A scheme integrated with deterministic-stochastic subspace system identification and the method of damage localization vector for damage detection of structures is considered and verified based on seismic response data in this study. Both numerical simulation and experimental verification have been carried out to verify the feasibility of the proposed scheme. A series of shaking table tests using a five-storey steel frame has been conducted in National Center for Research on Earthquake Engineering (NCREE), Taiwan. Both single and combinations of multiple damage conditions at various locations have been considered. Either a full or partial observation conditions have been taken into account in the system identification process. This study gives further insights into the scheme in terms of effectiveness, robustness, and limitation for damage localization of frame systems. Based on the numerical simulation and test results, the conclusions are drawn as the following.

- The N4SID algorithm for deterministic-stochastic models is more robust to noise contamination than the SRIM algorithm for purely deterministic models. With the first three modes of the shear-type frame system accurately identified from noise-contaminated signals with 10% NSR by using the N4SID and the flexibility-based DLV method, all damaged conditions considered can be successfully predicted.
- Damage localization utilizing seismic response data, in particular the floor accelerations, proves feasible not only analytically but also experimentally.
- Under a full observation condition where all floor responses are observed, the damaged location(s) can be successfully identified, regardless of single or multiple damage conditions.
- Under a partial observation condition where 3 out of 5 floor responses are observed, only those with single damage can be identified if the damaged storey is colocated with one of the observed floors. The scheme fails, however, to fully locate the damages in multiple damage conditions in general.
- The scheme proves to be sufficient in identifying new or extended damage(s) without exception under a full observation condition.

## Figures and Tables

**Table 1:** Parameters of a five-storey shear frame.

Structural parameter					
Storey	1F	2F	3F	4F	5F
*m*_*i*_ (kg)	25.8	25.8	25.8	25.8	25.8
*k*_*i*_ (MN/m)	39240	39240	39240	39240	39240

Modal parameter					
Mode	1	2	3	4	5
Frequency (Hz)	9.24	30.74	56.37	74.99	87.44
Damping ratio (%)	2.00	2.00	2.00	2.00	2.00

**Table 2:** Summary of damage assessment under various noise levels (numerical).

Case	D1	D2	D3	D4	D5	D13	D15	D135
nWSI_*j*_ (NSR = 0%)								
1F	**0.01**	0.46	0.47	1.00	1.00	**0.01**	**0.03**	**0.01**
2F	0.28	**0.02**	0.38	0.59	0.58	0.52	0.54	1.00
3F	0.96	1.00	**0.03**	0.63	0.36	**0.03**	1.00	**0.01**
4F	0.34	0.88	1.00	**0.02**	0.18	1.00	0.53	0.48
5F	1.00	0.78	0.92	0.26	**0.01**	0.98	**0.02**	**0.03**

nWSI_*j*_ (NSR = 10%)								
1F	**0.05**	0.80	1.00	1.00	1.00	**0.04**	**0.01**	**0.07**
2F	1.00	**0.09**	0.72	0.62	0.77	1.00	0.28	1.00
3F	0.30	0.74	**0.08**	0.85	0.66	**0.08**	1.00	**0.15**
4F	0.22	0.80	0.65	**0.02**	0.30	0.34	0.72	0.89
5F	0.39	1.00	0.35	0.46	**0.04**	0.38	**0.02**	**0.13**

**Table 3:** Summary of damage assessment w/full observation (experimental).

nWSI_*j*_^#^						
Case	M1	S1	S1M2	S12	S12M3	S123
1F	**0.09**	**0.05**	**0.04**	**0.07**	**0.01**	**0.04**
2F	1.00	0.44	**0.12**	**0.06**	**0.01**	**0.01**
3F	0.97	0.90	1.00	0.81	**0.11**	**0.04**
4F	0.72	1.00	0.89	0.90	0.51	0.83
5F	0.86	0.56	0.45	1.00	1.00	1.00

Performance∗	Good	Good	Good	Good	Good	Good

^#^nWSI_*j*_ ≤ 0.1 indicates serious damage; 0.1 < nWSI_*j*_ ≤ 0.2 indicates moderate damage.

∗Good indicates the damaged location(s) being identified without miss-judgment.

Fair indicates the damaged storey being identified but the extent might be underestimated.

Poor indicates failing to identify one of the damaged stories.

Fail indicates failing to identify more than one of the damaged stories.

**Table 4:** Summary of damage assessment w/partial observation (experimental).

nWSI_*j*_						
Case	M1/135	S1/135	M1/124	S1/124	S1M2/124	S1M2/125
1F	**0.14**	**0.11**	**0.17**	**0.16**	0.41	0.41
2F	—	—	1.00	1.00	1.00	1.00
3F	0.74	0.72	—	—	—	—
4F	—	—	0.41	0.43	0.33	—
5F	1.00	1.00	—	—	—	0.38

PMC	Good	Fair	Good	Fair	Fail	Fail

nWSI_*j*_						
Case	S1M2/1235	S12/124	S12/125	S12/1235	S12M3/1235	S123/1235

1F	**0.02**	0.96	1.00	**0.01**	**0.09**	**0.14**
2F	0.24	1.00	0.27	**0.20**	0.70	**0.15**
3F	1.00	—	—	1.00	1.00	0.48
4F	—	0.07	—	—	—	—
5F	0.66	—	0.81	0.78	0.63	1.00

PMC	Poor	Fail	Fail	Fair	Fail	Poor

**Table 5:** Damage assessment of ill-conditioned structures (experimental).

nWSI_*j*_					
Case	S1/M1	S1M2/S1	S12/S1M2	S12M3/S12	S123/S12M3
1F	**0.16**	**0.10**	0.26	0.24	0.32
2F	0.46	**0.01**	**0.03**	0.46	0.55
3F	1.00	0.29	0.36	**0.16**	**0.17**
4F	0.58	0.85	0.73	1.00	1.00
5F	0.90	1.00	1.00	0.62	0.72

PMC	Good	Good	Good	Good	Good

nWSI_*j*_					
Case	S12/M1	S12M3/M1	S123/M1	S12/S1	S123/S1

1F	**0.01**	**0.01**	**0.02**	0.48	1.00
2F	**0.02**	**0.02**	**0.01**	**0.07**	**0.03**
3F	0.13	**0.05**	**0.10**	0.35	**0.08**
4F	0.72	0.21	0.67	0.43	0.34
5F	1.00	1.00	1.00	1.00	0.69

PMC	Good	Good	Good	Good	Good
